# Transient binding facilitates super‐resolution imaging of functional amyloid fibrils on living bacteria

**DOI:** 10.1002/pro.70398

**Published:** 2025-12-23

**Authors:** Daniel J. Foust, Divya Kolli, Kailyn Jessel, Zeyang Hu, Matthew R. Chapman, Julie S. Biteen

**Affiliations:** ^1^ Department of Chemistry University of Michigan Ann Arbor Michigan USA; ^2^ Department of Molecular, Cellular, and Developmental Biology University of Michigan Ann Arbor Michigan USA

**Keywords:** biofilm, CsgA, curli, *Escherichia coli*, fluorescence correlation spectroscopy, fluorescence microscopy, Nile blue, points accumulation for imaging in nanoscale topography, single‐molecule localization microscopy, super‐resolution optical fluctuation imaging

## Abstract

Curli, which are the major proteinaceous components of the *Escherichia coli* biofilm extracellular matrix, help protect cells against environmental stressors, including dehydration and antibiotics. Composed of the amyloid proteins CsgA and CsgB, curli self‐assemble as these protomers are secreted into the extracellular space. The mechanisms of curli assembly and their functional roles within the extracellular matrix are incompletely understood. High‐resolution imaging tools compatible with live‐cell conditions provide a critical means to investigate the assembly and function of curli in their native context. In this study, we use super‐resolution imaging to visualize curli fibrils on living bacterial cells. Transient amyloid binding of the fluorogenic dye Nile blue facilitates two complementary super‐resolution fluorescence microscopy approaches: single‐molecule imaging via points accumulation for imaging in nanoscale topography and super‐resolution optical fluctuation imaging via pixel‐wise autocorrelation. Additionally, imaging fluorescence correlation spectroscopy was used to measure the characteristic relaxation times associated with Nile blue binding to CsgA fibrils. Together, these approaches offer a framework for imaging‐based biophysical characterization of curli structures on living cells.

## 1 INTRODUCTION

*Escherichia coli* and other enteric bacteria secrete amyloid proteins that self‐assemble into β‐sheet‐rich fibrils called curli (Chapman et al. 2002). Curli are the most abundant component of the *E. coli* extracellular matrix (ECM) that forms when bacterial communities form biofilms (McCrate et al. 2013). Bacterial biofilms contribute to disease pathogenesis and help confer resistance to environmental stressors, including antibiotics (Kikuchi et al. 2005; Van Gerven et al. 2018). Additionally, curli have been linked to Parkinson's disease pathology in mice, suggesting that they may play a role in potentiating neurodegenerative disease through interactions with host amyloids (Bhoite et al. 2022; Friedland and Chapman 2017; Sampson et al. 2020).

The amyloid protein CsgA is the major structural component of curli (Olsén et al. 1993). A second amyloid protein, CsgB, aids nucleation and anchoring of curli to the outer membrane (Bian and Normark 1997; Hammer et al. 2007). Fibrils, composed primarily of CsgA, form a meshwork surrounding the cells to provide a protective barrier (Ledvina et al. 2025). In addition to CsgA and CsgB, a set of accessory proteins regulates the intracellular transcription, transport, and conformation of the amyloids (Evans and Chapman 2014). The chaperone CsgC inhibits the amyloid fold and prevents intracellular fibrillization (Evans et al. 2015). CsgG forms an octameric pore to facilitate secretion across the outer membrane (Robinson et al. 2006). CsgE and CsgF support secretion through interactions with intracellular and extracellular surfaces of the pore, respectively (Klein et al. 2018; Nenninger et al. 2009; Swasthi et al. 2023). Curli production is tuned to a multitude of environmental factors, including temperature, osmolarity, and nutrient availability (Andreasen et al. 2019). Understanding the structure and function of curli fibers within the extracellular matrix of living cells is crucial for elucidating their roles in both bacterial physiology and human health.

In this study, we extend advanced fluorescence microscopy techniques to the novel application of characterizing curli fibers surrounding living cells. The fluorogenic dye Nile blue has recently been shown to transiently bind amyloid fibers in vitro (Ruiz‐Arias et al. 2022). Transient amyloid binding (TAB) has been exploited to facilitate single‐molecule localization microscopy (SMLM) in an approach known as points accumulation for imaging in nanoscale tomography (PAINT) (Sharonov and Hochstrasser 2006; Spehar et al. 2018; Sun et al. 2024). SMLM is based on the retrieval of the positions of single molecules from an image time series using algorithms to detect and localize single molecules (Tuson and Biteen 2015). Here, we have extended TAB‐PAINT for SMLM to image intact curli assemblies on living cells.

Single‐molecule detection of Nile blue is enabled by its solvatochromatism. Nile blue has reported quantum yields of 0.01 and 0.27 in water and methanol, respectively (Jose and Burgess 2006). In our experiments, this sensitivity manifests as an increase in brightness upon fluorophore binding in the nonpolar amyloid environment. With absorption and emission maxima of 626 and 668 nm in polar solvents, respectively, Nile blue can be used to image live cells with minimal cell damage and autofluorescence (Jose and Burgess 2006). In contrast, thioflavin T, another amyloid‐binding dye that has been demonstrated for TAB‐PAINT (Spehar et al. 2018), has peak absorption at 450 nm when bound to amyloid fibrils (Naiki et al. 1989), and this spectral region suffers from high autofluorescent backgrounds in cellular imaging (Shcherbakova et al. 2012).

SMLM encounters a limitation when the density of the target structure is highly variable, as is often the case *in cellulo*: under these conditions, it is challenging to tune the labeling strategy to effectively sample the entire structure. In a typical TAB‐PAINT realization of SMLM, the TAB fluorogen concentration needs to be optimized to enable the detection of isolated fluorescent molecules in each imaging frame. However, in variable environments, TAB fluorogen concentrations that are calibrated to efficiently sample low‐density regions, for example, isolated fibrils, will result in high‐density regions being oversampled such that single‐molecule detection is compromised. Conversely, if the fluorogen concentration is optimized for imaging high‐density regions, the low‐density regions will be undersampled, requiring prohibitively long acquisition times to obtain an image that captures the full extent of the structure.

We therefore considered an alternative to TAB‐PAINT by implementing a second, complementary approach: super‐resolution optical fluctuation imaging (SOFI) (Dertinger et al. 2009). SOFI is based on the pixel‐wise computation of cumulants to quantify correlations in the temporal fluctuations of the fluorescence signal (Dertinger et al. 2009). SOFI achieves increased image resolution based on the principle that the fluorescence from independent emitters is incoherent, and therefore, their contributions to the final image are uncorrelated. SOFI does not rely on the detection and isolation of signals from single molecules, but it does require that the target repeatedly switches between states with distinguishable fluorescence intensities to ensure adequate spatial sampling (Antarasen et al. 2024; Dertinger et al. 2009).

An additional benefit of SOFI is that imaging parameters can be chosen so that the data are also compatible with imaging fluorescence correlation spectroscopy (iFCS) (Kisley et al. 2015). iFCS is a camera‐based analog of fluorescence correlation spectroscopy (FCS) that calculates autocorrelation functions for each pixel to make thousands of measurements in parallel (Cooper and Harris 2014). We implemented iFCS to measure characteristic fluorescence fluctuation relaxation times associated with Nile binding to curli on living cells. Our measurements support the presence of two characteristic fluctuation times for Nile blue binding, which may indicate multiple binding modes or multi‐step binding, as has been demonstrated for other dye/amyloid pairs with confocal‐based FCS (Sarkar et al. 2023).

## 2 RESULTS

### 2.1 Nile blue stains *E. coli* cells expressing curli

We sought a labeling strategy compatible with fluorescence imaging of curli fibers on living *E. coli* cells while leveraging the capabilities of super‐resolution microscopy. We characterized Nile blue, a solvatochromic and fluorogenic dye that has been shown to transiently bind other amyloids, such as A*β*42 (Ruiz‐Arias et al. 2022). Additionally, its excitation and emission in the red portion of the electromagnetic spectrum make it well‐suited for low‐background imaging *in cellulo* because the cellular autofluorescent background is higher in the blue region of the spectrum (Shcherbakova et al. 2012).

**FIGURE 1:**
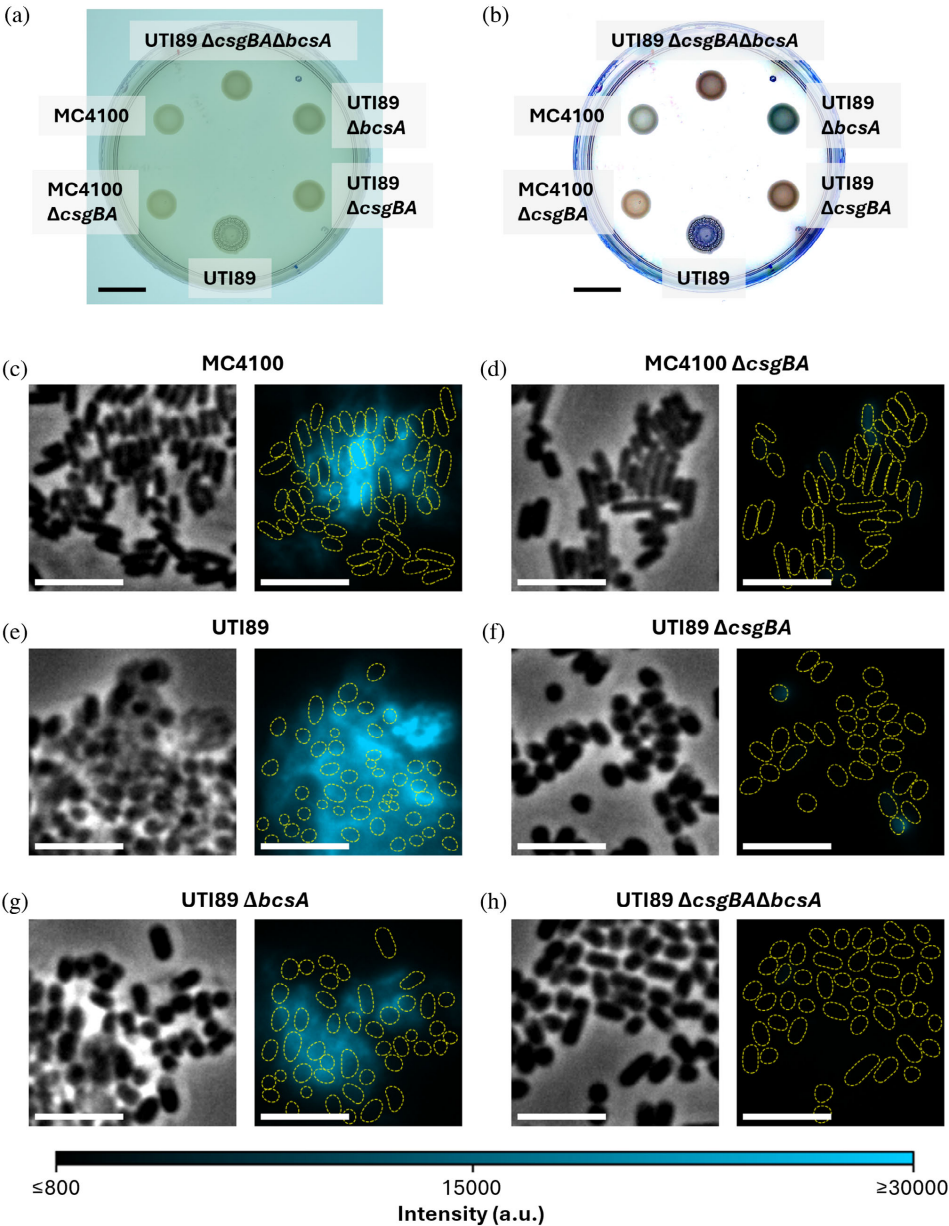
Nile blue stains *E. coli* cells that express curli. (a, b) *E. coli* biofilms grown on YESCA agar plates supplemented with Nile blue. (a) shows an unprocessed image captured with a smartphone camera. (b) Shows the processed image after background subtraction and color rebalancing to emphasize the difference in hue between the curli‐expressing strains and the curli‐deletion strains. Scale bars: 10 mm. (c–h) *E. coli* cells dispersed from biofilms grown on YESCA plates without Nile blue. 5 μM Nile blue was added after dispersion. Left images show phase contrast and right images show fluorescence detected under 640‐nm excitation. Yellow dashed lines indicate cell outlines segmented from the phase‐contrast images. Scale bars: 5 μm.

To test the affinity of Nile blue for the curli‐producing *E. coli* strains MC4100 and UTI89, we supplemented agar plates with 100 μM Nile blue and cultured cells for 72 h at 26°C. In these conditions, wild‐type cells form biofilms rich in mature curli. These biofilms exhibited a blue hue consistent with Nile blue absorbing red light, demonstrating the ability of the films to accumulate Nile blue from the agar (Figure 1a,b). In contrast, mutant strains MC4100 Δ*csgBA* and UTI89 Δ*csgBA*, both deficient in curli production, form biofilms with a severely diminished capacity to retain the dye (Figure 1a). This color difference is unremarkable when viewed with the unaided eye (Figure 1a), but is readily apparent after image processing to emphasize the difference in hues (Figure 1b). We also considered whether Nile blue has an affinity for cellulose, the other major proteinaceous component of *E. coli* biofilms (McCrate et al. 2013). A strain deficient for cellulose production, UTI89 Δ*bcsA,* showed a much stronger ability to accumulate Nile blue in comparison to its curli‐deficient counterpart, UTI89 Δ*csgBA*Δ*bcsA*. Notably, the presence of Nile blue in the agar did not prevent the growth of any strain tested, indicative of compatibility with live‐cell assays.

We visualized cells that had been grown on agar plates without Nile blue using epifluorescence microscopy. In these experiments, we instead added Nile blue to resuspended and partially dispersed cells just before pipetting them onto agarose pads for imaging. Under 640 nm excitation, we observed strong fluorescence in the curli‐expressing strains MC4100, UTI89, and UTI89 Δ*bcsA* (Figure 1c,e,g), in contrast to the curli‐deletion strains MC4100 Δ*csgBA*, UTI89 Δ*csgBA*, and UTI89 Δ*csgBA*Δ*bcsA* (Figure 1d,f,h). Nile blue fluorescence was more prominent in intact cell clusters that were more resistant to dispersion, and often entirely absent from singlet cells, consistent with the role of curli in maintaining the structural integrity of biofilms. We did observe fluorescence from a subset of cells in curli‐deletion strains, but the intensity was typically much dimmer and limited to the cell bodies.

### 2.2 Transient binding of Nile blue enables live‐cell super‐resolution imaging

**FIGURE 2:**
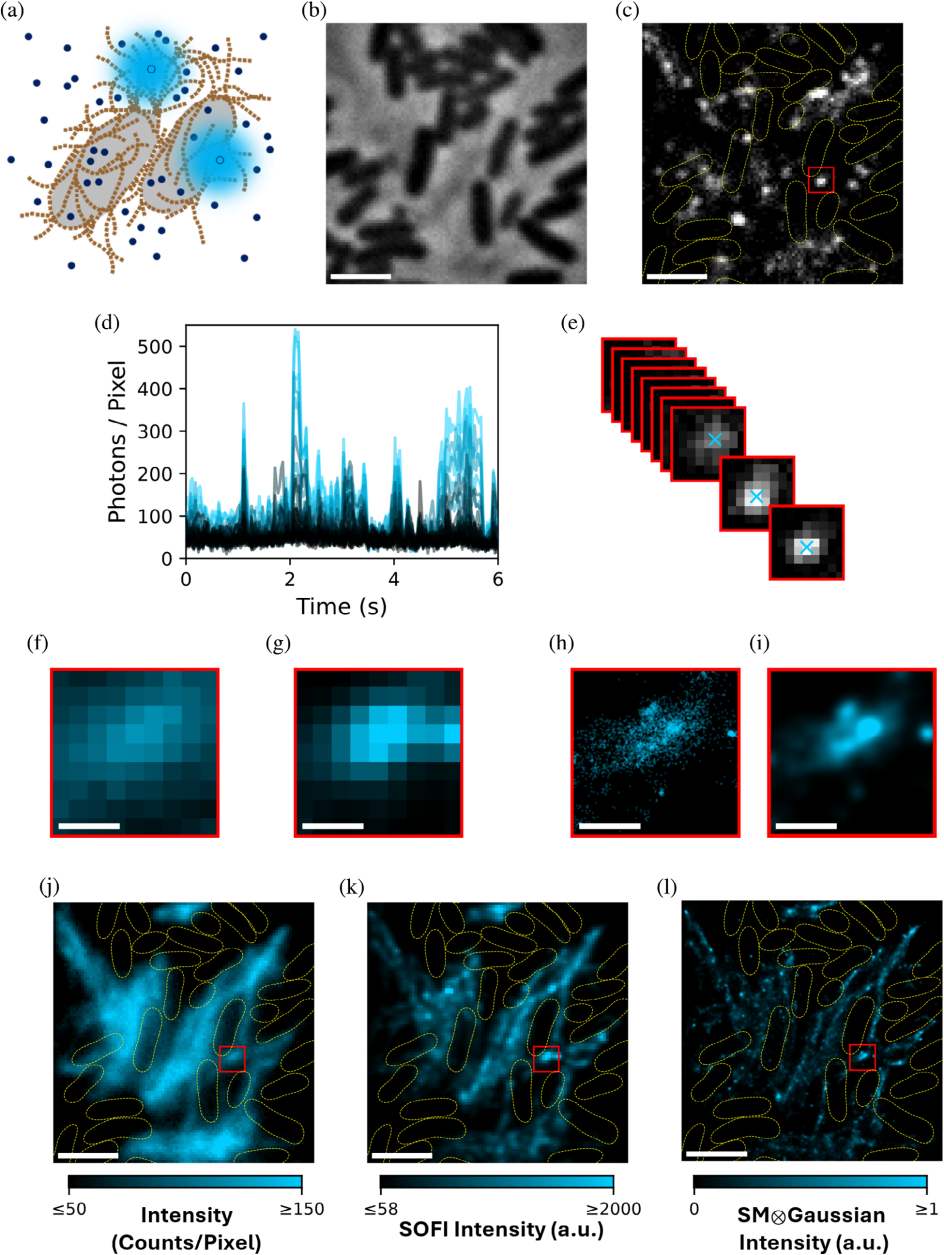
Transient amyloid binding enables PAINT and SOFI on living *E. coli* strain MC4100. (a) Schematic representation of two *E. coli* cells (light gray) expressing extracellular curli (light brown). Unbound Nile blue molecules (dark blue) remain in a dark state, while curli‐bound Nile blue molecules (light blue) fluoresce brightly. (b) Phase contrast image of *E. coli* cells surrounded by curli. (c) Single frame from a movie with 15 nM Nile blue in the agarose and imaging buffer under 640‐nm excitation. Dotted yellow lines indicate cells segmented from (b). (d) Temporal fluorescence intensity traces for a subset of the pixels within the red box in (c). Traces are colored according to the recovered SOFI intensity for each pixel. Six seconds of a 300‐s movie are shown. (e) Subpixel localization of molecules within the boxed region in (c). Blue crosses indicate molecule positions found by fitting to a 2D Gaussian. (f) Temporal average of red boxed region in (c). (g) Recovered SOFI intensities within the boxed region in (c). (h) Scatter plot of subpixel localizations found with the region of interest. (i) 2D histogram of localizations in (h) blurred by convolution with a Gaussian kernel with *σ* = 25 nm. (j) Temporal average of the entire field of view. (k) SOFI reconstruction for the entire field of view. (l) SMLM reconstruction for the entire field of view. (f, g, i) are on the same color scales as (j–l), respectively. Scale bars for (b, c) and (j–l) are 2 μm. Scale bars for (f–i) are 300 nm. Pixel size in (e) is 92 nm × 92 nm. Scales in (j, k) reflect measurements done in photon number‐resolving mode of the qCMOS camera.

We investigated the potential for transient amyloid binding (TAB) of Nile blue to facilitate super‐resolution fluorescence microscopy of curli on living *E. coli* (Figure 2). In these experiments, Nile blue was added to the cell medium (Figure 2a). Cells were identified using phase contrast microscopy (Figure 2b). Under 640 nm excitation, Nile blue brightens when bound to curli (Figure 2a,c and Movie S1, Supporting Information). Movies recorded with high temporal resolution (Figure 2c and Movie S1), Δ*t*_frame_ = 30 ms, were analyzed by pixel‐wise analysis of intensity fluctuations as in SOFI (Figure 2d), or by detection and subpixel localization of individual molecules as in PAINT (Figure 2e). The data in Figure 2 were collected with 12 nM Nile blue added to the agarose pad and imaging buffer.

The red‐boxed region in Figure 2c is used to illustrate both approaches. Pixels in which larger intensity fluctuations are observed have stronger measured autocorrelations (Figure 2d), which result in a resolution enhancement in comparison to a simple temporal average of all frames (Figures 2f,g and S1). In PAINT, the subpixel position of detected single molecules is determined frame‐by‐frame (Figure 2e). The output is a list of molecule positions (Figure 2e,h). We used the list of positions to generate a 2D histogram of molecule positions and convolved that histogram with a 2D Gaussian to generate the reconstructed image (Figure 2i).

Figure 2j–l shows the temporal average, SOFI reconstruction, and PAINT reconstruction for the full field of view. The TAB‐SOFI image, based on the calculation of second‐order cumulants, provides a modest resolution improvement (Figure S1), allowing us to more clearly visualize linear structures extending away from the cell bodies (Figure 2k). We measured the widths of isolated fibrils by fitting Gaussian functions, finding a median standard deviation, *σ*, of 113 nm in TAB‐SOFI images in comparison to 136 nm in diffraction‐limited temporal averages (Figure S1).

The TAB‐PAINT image provides the highest resolution image (Figures 2l and S2). Based on Fourier ring correlation, the empirically determined resolutions in our PAINT reconstructions are typically 30–50 nm (Figure S2). Some of the brightest regions in the temporal average are relatively dim in the TAB‐PAINT reconstruction, indicating that single‐molecule detection was compromised in these regions due to overlapping contributions from individual emitters (Figure 2c and Movie S1). Relatedly, we observe a degradation in the apparent resolution of TAB‐PAINT reconstructions as the Nile blue concentration is increased (Figure S2). Notably, the rate of detected single‐molecule binding events was very stable over 5‐min acquisition periods (Figure S3). Under our imaging conditions, we detected 690 ± 380 photons per molecule per frame (mean ± SD) (Figure S4).

**FIGURE 3:**
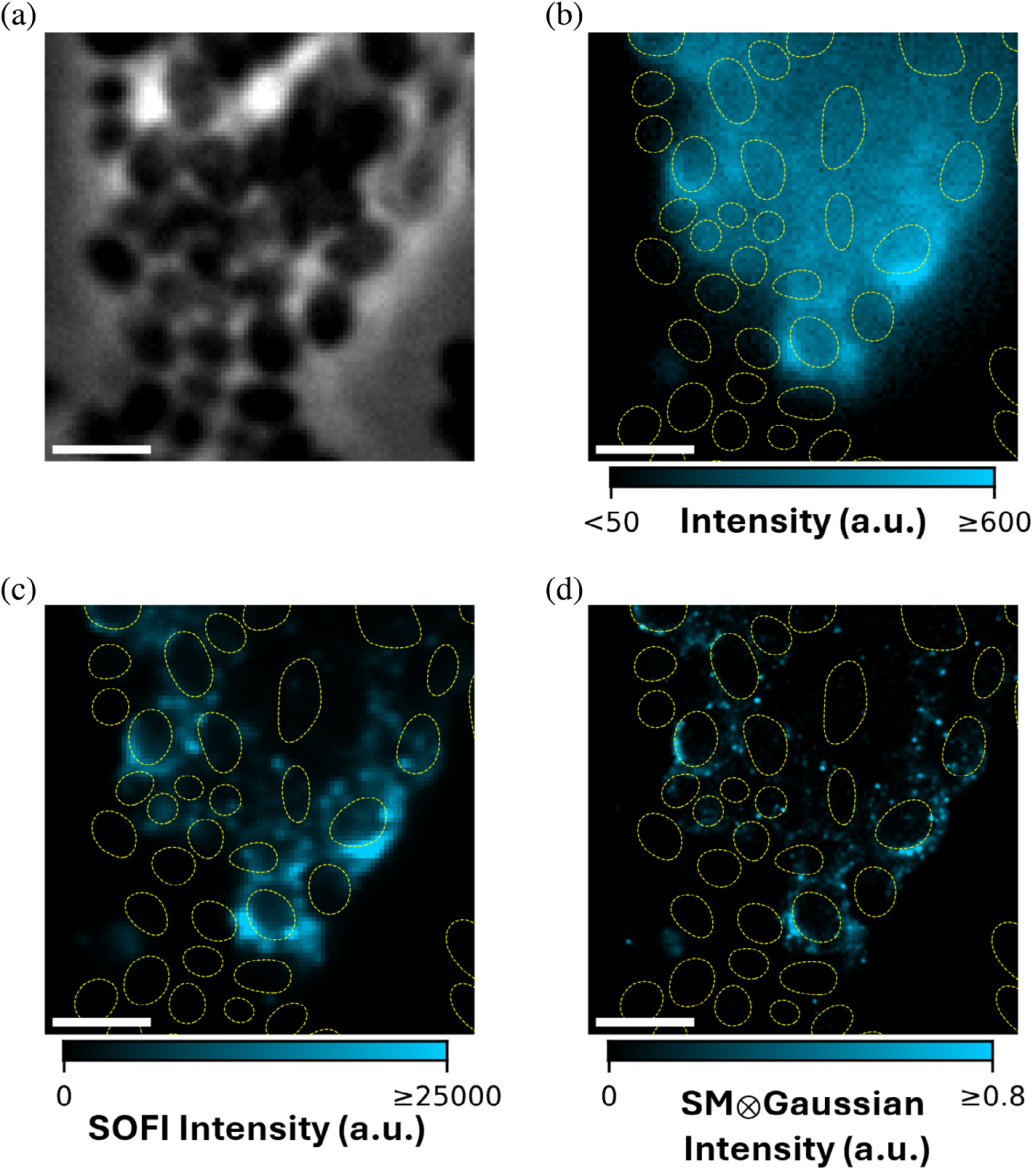
TAB‐SOFI and TAB‐PAINT with 1 nM Nile blue on pathogenic *E. coli* strain UTI89. (a) Phase contrast microscopy. (b) Temporal average of fluorescence. (c) SOFI microscopy. (d) PAINT microscopy. Dotted yellow lines indicate segmented cells and other relatively dark features in the phase contrast image. Scale bars: 2 μm. Scales in (b, c) reflect measurements made with the standard mode of the qCMOS camera.

Figure 3 shows TAB‐SOFI and TAB‐PAINT with 1 nM Nile blue applied to UTI89 cells (Figure 3a). The super‐resolution reconstructions allow us to more keenly observe differences in the spatial distribution of curli between the two strains (Figure 3b–d). In MC4100, we observe more linear structures extending away from the cells (Figure 2k,l), whereas in UTI89, curli are more limited to halos that fill the spaces immediately surrounding the cells (Figure 3c,d). We are also able to appreciate the optical sectioning inherent to both super‐resolution techniques (Figure 3c,d), which is poor in standard epifluorescence microscopy (Figure 3b). In SOFI, this feature is a consequence of molecules binding structures nearer to the focal plane generating larger fluctuations in comparison to out‐of‐focus molecules. Similarly, in PAINT, molecules binding in focus are more efficiently detected and localized. The sectioning allows us to appreciate that Nile blue binding is more prominent at the periphery of the large intact cluster of UTI89 cells (Figure 3c,d).

### 2.3 iFCS of Nile blue binding to curli surrounding live *E. coli*

The operating principle of SOFI is to leverage pixel‐wise analysis of fluorescence fluctuations to generate super‐resolution images from measured autocorrelations (Dertinger et al. 2009). A natural extension of this approach is to further investigate the temporal dependence of these fluctuations by analyzing autocorrelation functions (Kisley et al. 2015). Developed originally for the analysis of signals from single detectors (Magde et al. 1972), fluorescence correlation spectroscopy (FCS), when applied to camera‐based systems, is referred to as imaging FCS (iFCS) (Krieger et al. 2015).

**FIGURE 4:**
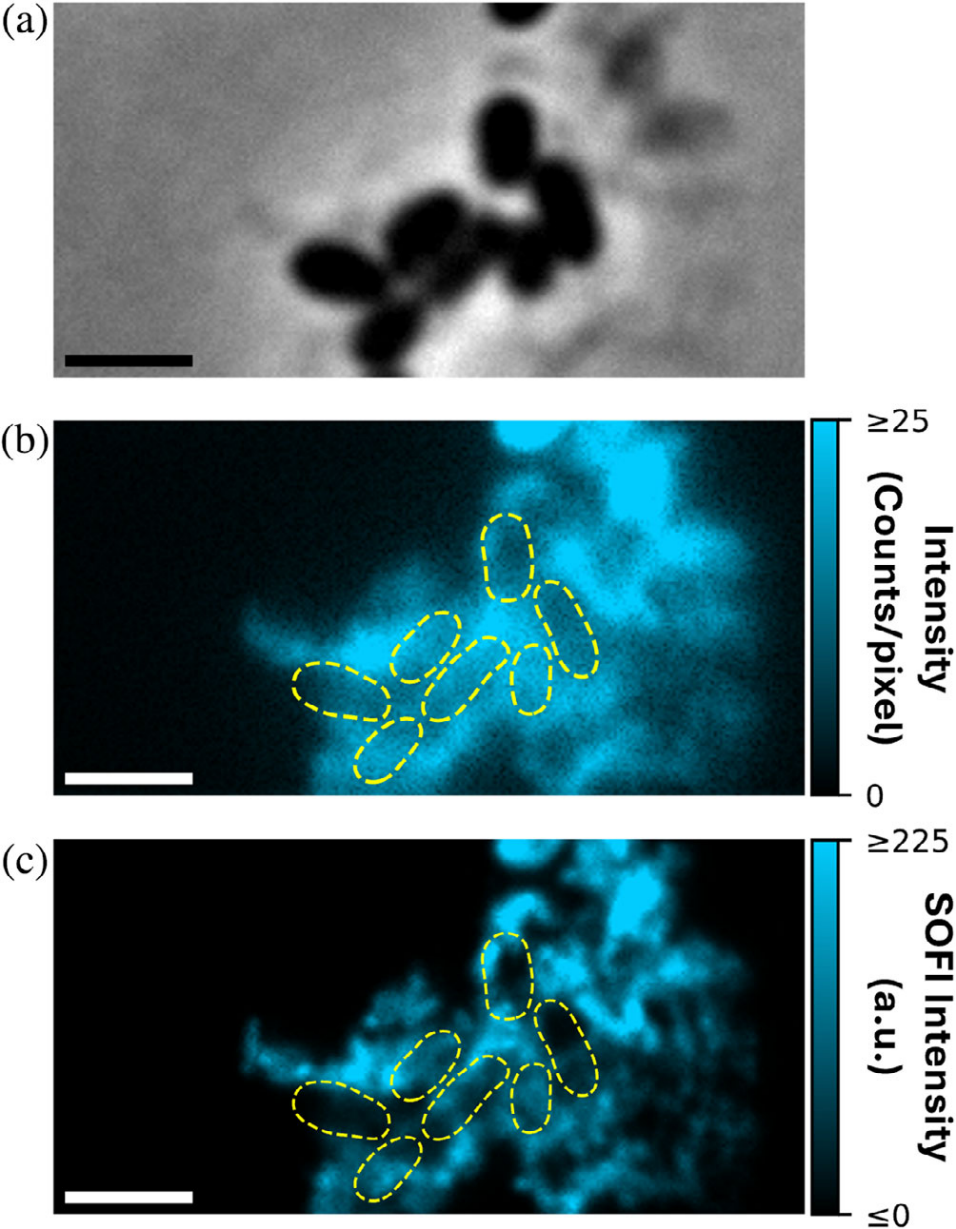
Super‐resolution optical fluctuation imaging via transient binding of Nile blue. (a) Phase‐contrast image of dispersed MC4100 *E. coli*. (b) Temporal average of Nile blue fluorescence. 5 μM Nile blue was added to the imaging buffer, but not the agarose pad. (c) SOFI reconstruction. Scales in (b) and (c) reflect measurements done in photon number‐resolving mode of the qCMOS camera. Dashed yellow outlines indicate cell positions determined by segmentation of the phase‐contrast image in (a). Scale bars: 2 μm.

To capture high temporal resolution fluorescence fluctuations from Nile blue binding to curli fibers on living *E. coli* cells, we collected movies with short exposure times, Δ*t*_frame_ = 2.5 ms (Movie S2). We used these movies to generate super‐resolved images of curli fibers via a second‐order SOFI reconstruction. Data collected at this higher temporal resolution is not suitable for PAINT due to the lower photon counts per frame. In contrast to the diffraction‐limited temporal average (Figure 4b), the SOFI map (Figure 4c) reveals the dense, irregular extracellular meshwork formed by curli with much greater detail. We used a much higher concentration of Nile blue, 5 μM, for these experiments; however, none was added to the agarose pad, such that the effective concentration was lower due to absorption into the pad. Nonetheless, the higher concentration enabled effective sampling of entire structures in 90 s acquisitions compared to the 5 min acquisitions used in the TAB‐SOFI/TAB‐PAINT combination experiments described above (Movies S1 and S2).

**FIGURE 5:**
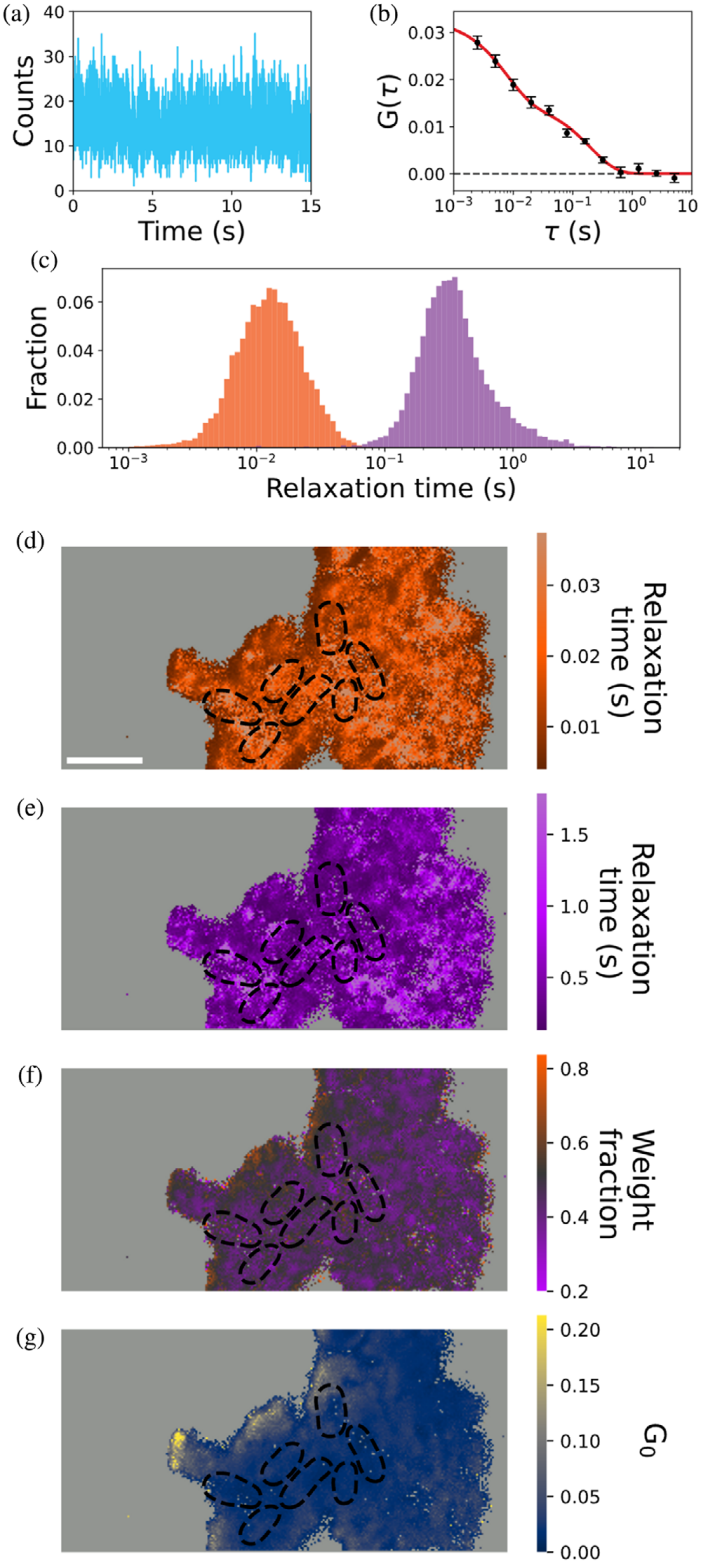
Imaging fluorescence correlation spectroscopy measures Nile blue kinetics. (a) Fluorescence intensity trace for a representative pixel from the same movie used to generate the SOFI image in Figure 4. (b) Average autocorrelation function for the same pixel over six 15 s movie segments calculated as a function of timelapse, *τ*. Black circles indicate the mean of the autocorrelation functions for that pixel, and error bars indicate the standard error of the mean. The red line is a fit to a two‐component binding model. (c) Histogram of characteristic relaxation times, determined by fitting the autocorrelation functions for each pixel with SNR > 10. Shorter and longer relaxation times are shown in orange and purple, respectively. (d–f) Maps of parameters obtained from fitting the autocorrelation function for each pixel. (d) Short relaxation times. (e) Long relaxation times. (f) Estimated fraction of molecules associated with the shorter relaxation time. (g) Amplitude of the autocorrelation function, *G*_0_. Black dashed lines indicate segmented cell outlines. Pixels with insufficient SNR for correlation analysis are colored light gray. Scale bars: 2 μm.

Furthermore, the fluorescence intensity traces provide information to characterize the relaxation times of Nile blue on curli using iFCS. To perform iFCS, we calculated the autocorrelation function from the temporal fluorescence intensity traces from each pixel (Figure 5a). We fit individual autocorrelation functions with model functions using least squares optimization (Figure 5b). Generally, a single‐component model was inadequate for fitting the autocorrelation functions, as evaluated using reduced‐*χ* ^2^ statistics. To better capture the observed dynamics, we used a two‐component model, yielding average fast and slow relaxation times of 23 ± 1 ms (mean ± SEM) and 482 ± 13 ms, respectively. The relaxation time distributions are shown in Figure 5c.

We use the general term relaxation time because the sources of the intensity fluctuations cannot be identified unambiguously. Previously, the presence of two components in point FCS measurements on fluorogenic dyes binding to amyloids has been used as evidence of two residence times, each associated with a distinct binding mode (Sarkar et al. 2023). Multimodal binding may also be characteristic of Nile blue binding to CsgA fibrils; however, we cannot exclude the possibility that the shorter relaxation time corresponds to blinking associated with a photophysical phenomenon (Midhun et al. 2023; Schenk et al. 2004).

iFCS offers advantages over classic single‐point FCS measurements by enabling thousands of measurements to be made in parallel and, consequently, by spatially resolving these measurements. Leveraging these advantages, we generated maps of characteristic binding times (Figure 5d,e), associated weight fractions (Figure 5f), and amplitudes, *G*_0_, of the fitted autocorrelation functions (Figure 5g). Weight fractions estimate the fraction of bound molecules associated with each relaxation time.

The relaxation times and their associated weight fractions do not generally follow any clear spatial organization relative to the positions of cells (Figure 5d–f), though we observed that both the fast and slow relaxation times are lower (Figure 5d,e) in the regions of dimmer fluorescence intensity (Figure 4b,c) that tend to be at the edges of the region of interest. This correspondence is most likely an artifact: autocorrelation function fitting with least‐squares optimization is known to be a biased estimator that underestimates relaxation times in photon‐poor regions (Kohler et al. 2023). Accordingly, the weight fraction associated with the shorter relaxation time is higher in these regions (Figure 5f).

To compare with iFCS results, apparent binding times from TAB‐PAINT experiments (Figures 2 and 3) were measured by finding the number of consecutive frames molecules were “on” (Figure S5). The distribution of “on” frames per detected molecule could not be described adequately with a monoexponential decay (Figure S5); a biexponential decay model provided a better fit, in agreement with iFCS, which measures two distinct binding times (Figure S5). The short decay time of 17 ms in TAB‐PAINT also agrees reasonably well with the 23 ms short relaxation time measured by iFCS. However, the longer decay time of 77 ms in TAB‐PAINT is much less than the 482 ms long relaxation time determined by iFCS, and the weight of this long binding time fraction in the TAB‐PAINT “on” times suggests that TAB‐PAINT is mainly sensitive to the shorter binding events. This discrepancy may be due to the dependence of TAB‐PAINT “on” times on the accurate linkage of detections in consecutive frames, and in these experiments, we did not optimize for faithful linkage of continuously “on” molecules across frames. Instead, overlap of neighboring emitters often caused interruptions in linking (Figure 2d and Movie S1). Blinking is another potential source of interruptions. Therefore, it is unsurprising that TAB‐PAINT was biased toward the shorter decay times.

## 3 DISCUSSION

We have demonstrated that the fluorogenic dye Nile blue is readily absorbed by *E. coli* expressing extracellular amyloid fibrils known as curli (Figure 1). Furthermore, we showed that transient binding of Nile blue is an effective tool for imaging curli on living cells (Figures 1, 2, 3, 4). A major advantage of TAB relative to other labeling strategies is that it does not require covalent modification of the protein of interest. This feature is especially important in the context of amyloids, as covalent modifications may alter their aggregation kinetics (Bhoite et al. 2023). We demonstrated that this flexible, non‐covalent labeling approach can be leveraged for both TAB‐PAINT (Figures 2 and 3) and TAB‐SOFI (Figures 2 and 4). Additionally, by coupling SOFI with iFCS, we measured autocorrelation functions that suggest two distinct relaxation times associated with Nile blue binding to CsgA fibrils (Figure 5).

SMLM and SOFI each have strengths and weaknesses. SMLM is capable of higher resolutions, as the precision with which single‐molecule positions can be determined is primarily limited by the number of detected photons (Mortensen et al. 2010). Typical SMLM resolutions are in the tens of nanometers (Sage et al. 2019). In SOFI, the achievable enhancements in resolution are proportional to the square root of the cumulant order (Dertinger et al. 2009). In this work, we used second‐order cumulants, theoretically providing a 1.4‐fold resolution enhancement, though our fibril width measurements suggest a more modest resolution enhancement in these experiments (Figure S1). Higher‐order cumulants can provide greater resolution, but they require more data and are more susceptible to artifacts (Basak et al. 2025), though a recent report leverages an artificial neural network with a transformer architecture to achieve SMLM‐like resolution using SOFI‐like inference from temporal correlations (Reinhard et al. 2025). Notably, these deep learning‐based approaches require additional effort up front to generate experimentally realistic labeled training data (Liu et al. 2024).

Another consideration in super‐resolution microscopy is the set of inherent trade‐offs in acquisition time, temporal resolution, and spatial sampling. The conventional SMLM detection and localization scheme used in this work requires that the emission patterns of single molecules be well‐separated on the camera for accurate determination of molecular positions. Although we were able to detect single molecules in both curli‐dense spaces and the sparser regions where individual fibers extend away from cells with 15 nM Nile blue (Figure 2l and Movie S1), the sampling of the structures was not necessarily proportional to the true number of binding events. In the most curli‐dense regions, the dye concentration was higher than optimal, and overlapping Nile blue binding events led to errors in single‐molecule detection and localization (Figure 2c and Movie S1). Conversely, the relatively low dye concentration required long acquisition times to adequately sample the more sparse regions. Sophisticated algorithms that support multi‐emitter fitting (Holden et al. 2011; Huang et al. 2011) or leverage neural networks trained to extract localizations from overlapping emitters (Nehme et al. 2018; Reinhard et al. 2025; Speiser et al. 2021) can partially alleviate these limitations in SMLM. In contrast, SOFI does not inherently necessitate limitations on the emitter density, but it does require that a sufficient number of independent fluctuations are observed (Antarasen et al. 2024). Well‐sampled reconstructions have been achieved using as few as 20 frames, and continued improvements in SOFI algorithms remain an area of active research (Antarasen et al. 2024; Basak et al. 2025; Zhao et al. 2023).

We have demonstrated that SMLM and SOFI can be applied to the same data (Figures 2 and 3), though it may be prudent in future applications to optimize imaging conditions for a single post‐acquisition analysis. SOFI is the better option when high temporal resolution or high throughput is desired, and when the additional resolution enhancement afforded by SMLM is not necessary. In this case, higher concentrations of the fluorogen may be used to rapidly sample the entire structure (Figure 4). In addition to providing better resolution, SMLM can be extended with polarization resolution to measure single‐molecule orientations, a technique known as single‐molecule orientation localization (Backlund et al. 2014). Single‐molecule orientations provide readouts of in situ structural biology. The orientation of Nile blue has been used to probe the amyloid A*β*42 (Sun et al. 2024). Such an approach could provide insight into the in vivo assembly of CsgA curli fibers. To optimize for SMLM, lower dye concentrations and longer acquisition times should be considered.

To our knowledge, this is the first application of iFCS to spatially resolve transient amyloid binding kinetics. CsgA fibrils are known to take on different conformations in response to environmental conditions (Andreasen et al. 2019; Bu et al. 2024). We posit that iFCS, in conjunction with amyloid‐sensitive fluorogens whose binding kinetics are sensitive to fibril structure, could be used to probe structural variations in intact biofilms. Recently, covalent labeling of CsgA with fluorescent tags has been used to quantify elongation rates in vitro (Olsen et al. 2024; Olsen et al. 2025a; Olsen et al. 2025b). It is not known whether these findings translate to living cells, where the presence of CsgB—the nucleation‐inducing curli protein—and the complexity of the cellular milieu may significantly alter assembly dynamics (Bian and Normark 1997; Hammer et al. 2007). The imaging approaches explored in this work present opportunities to probe curli assembly in vivo. A feature of TAB and other PAINT techniques that we have not explored in this work is the reduced susceptibility to signal loss due to photobleaching (Figure S3) (Jang et al. 2023). This property could be leveraged to facilitate long‐term imaging in support of quantifying fibril elongation and extracellular matrix development.

## 4 MATERIALS AND METHODS

### 4.1 Cell preparation

We cultured *E. coli* strains MC4100, UTI89, and their mutants by spotting 4 μL of liquid culture grown to saturation overnight in Luria‐Bertani broth onto agar plates containing yeast extract and casamino acids (YESCA). Plates were supplemented with 100 μM Nile blue where indicated. We grew cultures for 48–72 h at 26°C. Macroscopic images of agar plates were collected by illuminating from below with a white light source (Analytik Jena, UVP UV/white light transilluminator, PN 95‐0418‐01) and capturing photographs with a Samsung S22 smartphone. Images were processed using ImageJ.

We observed that cells grown on Nile blue‐supplemented plates exhibited a higher degree of nonspecific fluorescence under 640 nm excitation. Accordingly, for imaging dispersed cells, we grew cells on plates without Nile blue and added Nile blue just prior to preparation for imaging. To prepare cells for microscopy, we scooped a lentil‐sized portion (~3 mm diameter) of the biofilm using a disposable sterile loop and transferred it to 500 μL of phosphate‐buffered saline (PBS). We pipetted up and down to disperse the clump into single cells and small cell clusters.

For imaging, dispersed cells were deposited on agarose pads prepared by adding 2% w/v agarose to PBS and heating in a microwave until dissolved. 600 µL of molten agarose was deposited onto a coverglass, and a second coverglass was placed on top so that the agarose–glass interface formed a flat surface. To image, the top coverglass was removed, and 2–5 μL of cells were deposited and covered with a fresh #1.5H thickness coverglass (Thorlabs). For bulk imaging and iFCS experiments, 5 μM Nile blue was added to disperse cells immediately prior to deposition on an agarose pad. For combination TAB‐PAINT/TAB‐SOFI experiments, 1, 3, 12, or 25 nM Nile blue, as indicated, was added to the molten agarose as it cooled during the preparation of the pad, and a matching concentration was added to the cells immediately prior to deposition on the pad. If Nile blue was not added to the agarose pad, higher concentrations compensated for absorption into the pad. However, this effect was not noticeable in the form of decreasing signal levels over typical imaging times (~5 min).

### 4.2 Fluorescence microscopy

We imaged live *E. coli* samples on a custom‐built optical instrument configured for epifluorescence microscopy. To excite Nile blue, we used 640 nm continuous‐wave laser excitation (Coherent CUBE 640‐40C) cleaned up with a notch filter (Chroma, Z640). The beam was directed through the back aperture of an Olympus IX71 microscope and focused onto the back focal plane of a 100×, 1.45 numerical aperture objective lens (Olympus X‐Apo). We used a dichroic mirror (Chroma Di01‐R640) to direct collimated light onto the sample. A combination of linear polarizer (Thorlabs LPVISB‐050‐M), half‐wave plate (Thorlabs AHWP10M‐600), and liquid crystal variable retarder (Thorlabs LCC1223T‐A) was used to correct for the phase shift introduced by the dichroic mirror to produce circularly polarized light at the sample. The average power density within the full width at half maximum diameter of the Gaussian beam at the sample plane was approximately 10 μW/μm^2^. We collected emitted fluorescence with the same objective lens, filtered it by transmission through the dichroic mirror and a long pass filter (Semrock, BLP01‐635R‐25), and directed it onto an ORCA‐Quest qCMOS camera (Hamamatsu C15550‐20UP) positioned at the image plane of the IX71 tube lens. The pixel size at the object plane was 46 nm or 92 nm when 2 × 2 binning was used. We operated the camera in ultra‐quiet mode with photon number resolving for low‐intensity imaging (less than 200 photons/pixel/frame or 800 photons/pixel/frame with 2 × 2 binning) and switched off photon number resolving for higher‐intensity imaging due to the limited dynamic range.

For bulk imaging, single images were captured with 50 ms exposure times with reduced power, 1 μW/μm^2^ within the FWHM of the Gaussian beam. For combination TAB‐PAINT/TAB‐SOFI experiments, we recorded movies with 30 ms exposure times for 10,000 frames per movie. 2 × 2 pixel binning was used so that the pixel size at the object plane was 92 nm × 92 nm.

For iFCS‐SOFI experiments, a higher temporal resolution, 2.5 ms exposure time was used to capture more of the fast dynamics associated with Nile blue binding. The pixel size was 46 nm × 46 nm. 1.5 min acquisitions totaling 36,000 frames were divided into six 15 s segments for analysis.

### 4.3 Single‐molecule localization microscopy

We detected and localized single molecules using Palmari, a plugin for the Python‐based image analysis software Napari (Sofroniew et al. 2025). To detect single molecules, we applied a Laplacian‐of‐Gaussian filter (*σ* = 1.5 pixels) to raw movie frames (Godinez et al. 2009). We identified candidate molecules by applying a threshold to each filtered frame equal to three times the standard deviation of the filtered image pixel values. We fit sub‐images of single molecules with a 2D‐Gaussian PSF using GPUfit (Przybylski et al. 2017). We discarded candidate molecules if a high‐quality fit could not be achieved (Isaacoff et al. 2019).

We constructed super‐resolution density maps by generating 2D histograms of all localizations with 5 nm × 5 nm bins. We convolved these histograms with a 2D Gaussian kernel with *σ* = 25 nm. Empirical resolution measurements determined from our data using Fourier ring correlation suggest the effective full width at half maximum for our data is ~30–50 nm (Figure S1), corresponding to a *σ* ranging from ~12 to 20 nm. Therefore, we conservatively selected 25 nm for our Gaussian convolution kernel.

### 4.4 Super‐resolution optical fluctuation imaging

The principle of SOFI is to enhance spatial resolution by applying temporal correlation analysis to raw image time series. This approach leverages the fact that the fluorescence emission of unique emitters is uncorrelated. By performing pixel‐wise calculations of cumulants, individual emitter contributions are independent in the final image. We used second‐order cumulants to generate SOFI images,

(1) $$C_{2}\left(\tau ;x,y\right)={\left\langle ∆I\left(t;x,y\right)∆I\left(t+\tau ;x,y\right)\right\rangle }_{t},$$

where $\Delta I\left(t\right)=I\left(t\right)-{\left\langle I\left(t\right)\right\rangle }_{t}$. We summed second‐order cumulants for many time lags, *τ*, to generate SOFI images, $I_{x,y}^{\text{SOFI}}$,

(2) $$\begin{array}{c} I_{\text{SOFI}}\left(x,y\right)=\sum_{0<\tau \leq \tau_{\max}}C_{2}\left(\tau ;x,y\right) \end{array}.$$

For the higher temporal resolution acquisitions, Δ*t*_frame_ = 2.5 ms, we chose $\tau_{\max}$ to be the point at which the autocorrelation function had decayed by ~50% to avoid noise contributions from larger time lags. For lower temporal resolution acquisitions, we only used the first nonzero time lag.

### 4.5 Imaging fluorescence correlation spectroscopy

Using the same high temporal resolution movies used for generating SOFI images, Δ*t*_frame_ = 2.5 ms, we calculated the temporal autocorrelation function, $G\left(\tau ;x,y\right)$, from the intensity trace, $I\left(t;x,y\right)$, for each pixel,

(3) $$\begin{array}{c} G\left(\tau ;x,y\right)=\frac{{\left\langle \Delta I\left(t;x,y\right)\Delta I\left(t+\tau ;x,y\right)\right\rangle }_{t}}{{\left\langle I\left(t;x,y\right)\right\rangle }_{t}}. \end{array}$$

Notably, the numerator in Equation (3) is equivalent to Equation (1), allowing computations to be shared between SOFI and iFCS when applied to the same data.

We averaged autocorrelation curves from six 15 s movie segments and used the standard error of the mean (SEM) to quantify the uncertainty at each time lag.

We fit experimental autocorrelation functions, *G*(*τ*), with a two‐component binding model,

(4) $$G\left(\tau \right)=\begin{array}{l} G_{0}\left(1-f_{\text{fast}}+f_{\text{fast}}e^{-\tau /\tau_{\text{fast}}}\right)\cdot \left(1-f_{\text{slow}}+f_{\text{slow}}e^{-\frac{\tau }{\tau_{\text{slow}}}}\right)\\ +\;G\left(\infty \right). \end{array}$$

$G_{0}$ is the amplitude and is inversely proportional to the average number of molecules. $\tau_{\text{fast}}$ and $\tau_{\text{slow}}$ are binding relaxation times, and $f_{\text{fast}}$ and $f_{\text{slow}}$ are the corresponding fractions of bound molecules associated with each relaxation time. $G\left(\infty \right)$ is the offset.

We also considered a single‐component model that can be derived from Equation (4) by setting $f_{\text{fast}}=1$ and $f_{\text{slow}}=0$. We evaluated models by calculating reduced‐*χ* ^2^ statistics from residuals of the least squares optimization. Generally, we calculated reduced‐$\chi^{2}\gg 1$ from fitting the single‐component model, indicating an inadequate description of the data, and reduced‐$\chi^{2}\approx 1$ for the two‐component model.

To generate maps of the fit parameters, results from low signal‐to‐noise (SNR) autocorrelation functions were excluded. SNR was estimated by dividing the autocorrelation function amplitude by the standard deviation of the last five time lags of the autocorrelation function. Results from autocorrelation functions with SNRs below 10 are not shown on maps and are substituted with light gray pixels.

### 4.6 Fourier ring correlation

To estimate the effective resolution of TAB‐PAINT images, single‐molecule localizations were randomly assigned to one of two subsets. Each subset was used to generate a 2D histogram of localizations with 5 nm × 5 nm bins. The 2D Fourier transform of each histogram was calculated. Fourier space is divided into equally spaced concentric rings corresponding to bands of spatial frequencies. The normalized correlation within each ring was calculated according to

(5) $$\begin{array}{c} \mathrm{FRC}\left(r_{i}\right)=\frac{\sum_{r\in r_{i}}F_{1}\left(r\right)F_{2}{\left(r\right)}^{*}}{\sqrt{\left(\sum_{r\in r_{i}}{\left|F_{1}\left(r\right)\right|}^{2}\right)\left(\sum_{r\in r_{i}}{\left|F_{2}\left(r\right)\right|}^{2}\right)}}. \end{array}$$

$F_{1}\left(r\right)$ and $F_{2}\left(r\right)$ are the Fourier transformed histograms. $r_{i}$ is the set of pixels within ring i. r is a pair of pixel coordinates in 2D Fourier space. Asterisk (*) denotes complex conjugation. A threshold of 1/7 was used to find the critical frequency whose inverse corresponds to the effective resolution (Nieuwenhuizen et al. 2013).

## AUTHOR CONTRIBUTIONS

**Daniel J. Foust:** Conceptualization; investigation; writing – original draft; methodology; formal analysis; visualization; writing – review and editing. **Divya Kolli:** Methodology; investigation; writing – review and editing. **Kailyn Jessel:** Methodology; investigation. **Zeyang Hu:** Investigation. **Matthew R. Chapman:** Conceptualization; methodology; supervision; funding acquisition; project administration; writing – review and editing. **Julie S. Biteen:** Conceptualization; methodology; supervision; funding acquisition; project administration; writing – review and editing.

## Supporting information


**Figure S1.** Apparent widths of isolated filaments in temporal average and SOFI images.
**Figure S2.** Fourier ring correlation curves for TAB‐PAINT reconstructions.
**Figure S3.** Average number of molecules detected per frame in TAB‐PAINT experiments.
**Figure S4.** Distribution of molecule brightnesses for molecules detected in TAB‐PAINT experiments.
**Figure S5.** Distribution of “on” times for molecules detected in TAB‐PAINT experiments.

**Movie S1.** Single‐molecule localization microscopy via transient binding of Nile blue. A subset of frames from the movie that was used to create Figure 2. The 33‐fps playback speed is equal to the acquisition speed. Scale bar: 2 μm.

**Movie S2.** Super‐resolution optical fluctuation imaging via transient binding of Nile blue. A subset of frames from the movie that was used to create Figures 4 and 5. The 40‐fps playback speed is one‐tenth of the 400‐fps acquisition speed. Scale bar: 2 μm.

## Data Availability

The data that support the findings of this study are available from the corresponding author upon reasonable request.
