# Supplementary material for: Transient binding facilitates super‐resolution imaging of functional amyloid fibrils on living bacteria
Source: Protein Sci. 2025 Dec 23;35(1):e70398. doi: 10.1002/pro.70398 (PMC12723734; doi:10.1002/pro.70398)
Supplement: Supplementary file 1 — Figure S1. Apparent widths of isolated filaments in temporal average and SOFI images. Figure S2. Fourier ring correlation curves for TAB‐PAINT reconstructions. Figure S3. Average number of molecules detected per frame in TAB‐PAINT experiments. Figure S4. Distribution of molecule brightnesses for molecules detected in TAB‐PAINT experiments. Figure S5. Distribution of “on” times for molecules detected in TAB‐PAINT experiments. [file PRO-35-e70398-s003.pdf]

Supplementary materials:

Transient binding facilitates super-resolution imaging of functional amyloid fibrils on living bacteria

Daniel J. Foust[1], Divya Kolli[2], Kailyn Jessel[2], Zeyang Hu[1], Matthew R. Chapman[2], Julie S. Biteen[1]

1. Department of Chemistry, University of Michigan, Ann Arbor, MI, United States
2. Department of Molecular, Cellular, and Developmental Biology, University of Michigan, Ann Arbor, MI, United States

Supplementary Movie Captions

Movie S1

Single-molecule localization microscopy via transient binding of Nile blue. A subset of frames from the movie that was used to create Figure 2. The 33-fps playback speed is equal to the acquisition speed. Scale bar: 2 μm.

Movie S2

Super-resolution optical fluctuation imaging via transient binding of Nile blue. A subset of frames from the movie that was used to create Figures 4 – 5. The 40-fps playback speed is one-tenth of the 400-fps acquisition speed. Scale bar: 2 μm.

Supplementary Figures

Figure S1. Apparent widths of isolated filaments in temporal average and SOFI images.

Figure S2. Fourier ring correlation curves for TAB-PAINT reconstructions.

Figure S3. Average number of molecules detected per frame in TAB-PAINT experiments.

Figure S4. Distribution of molecule brightnesses for molecules detected in TAB-PAINT experiments.

Figure S5. Distribution of 'on' times for molecules detected in TAB-PAINT experiments.

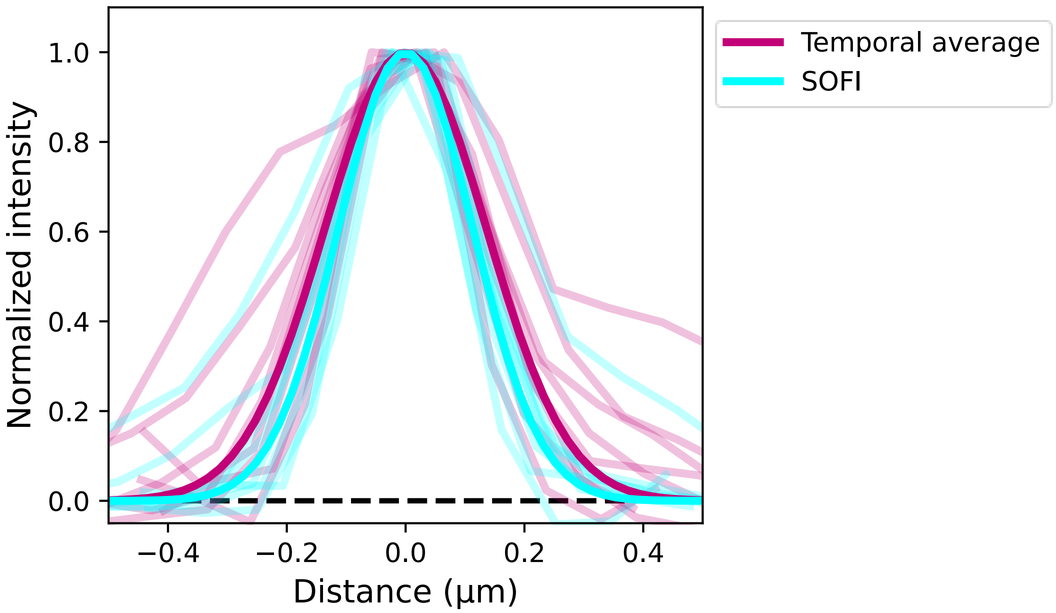

Figure S1. Apparent widths of isolated filaments in temporal average and SOFI images. Lines were drawn orthogonal to isolated linear structures. Line intensity profiles were fit with Gaussian functions to determine their widths. The same set of lines were used for temporal average and SOFI images. Semi-transparent lines are normalized individual line profiles. Opaque lines are Gaussian functions calculated using the median fitted standard deviation, σ, for each type of image. Median σ values were 0.136 µm and 0.113 µm for temporal average and SOFI images, respectively.

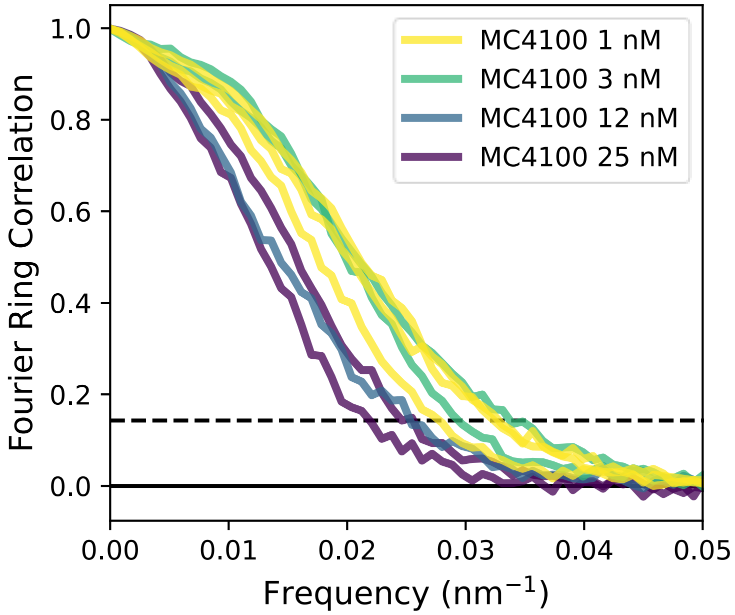

Figure S2. Fourier ring correlation curves for TAB-PAINT experiments. For each experiment, the same concentration of Nile blue was added to the agarose pad and surrounding media.

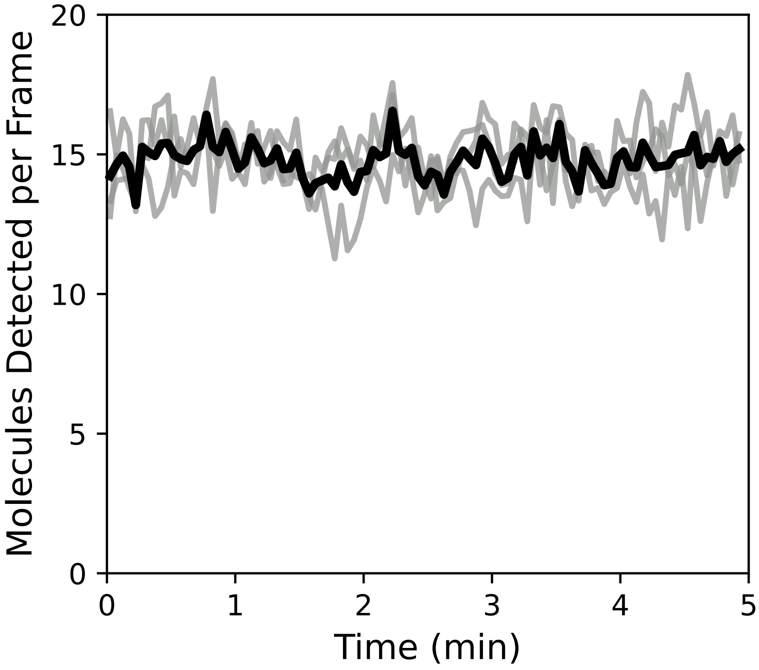

Figure S3. Average number of molecules detected per frame in TAB-PAINT experiments. Data are from three movies of 1 nM Nile blue on MC4100 cells. Frame times were 30 ms and the frame image size is 11.8 μm × 11.8 μm. The average number of molecules detected per frame was calculated for 100-frame segments for 10000 frames. Gray lines show the average number of molecules for individual movies. The black line is the average of the three movies.

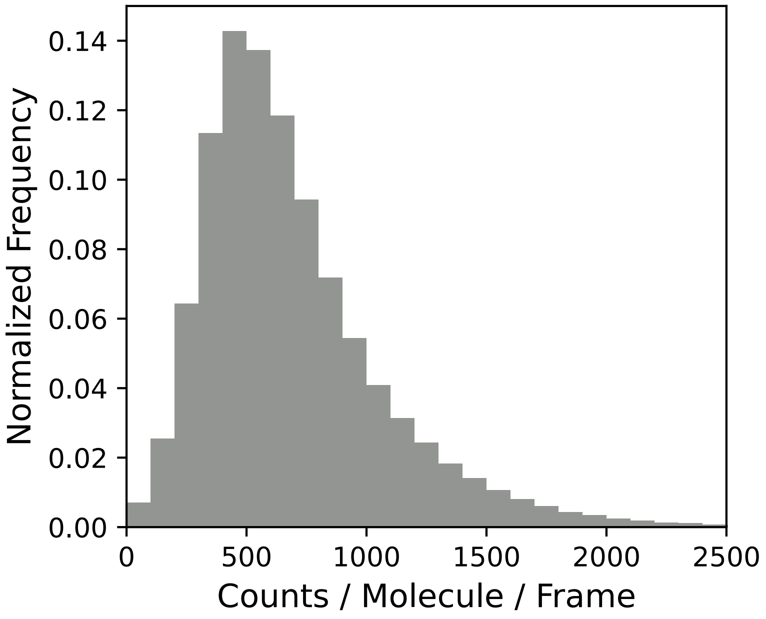

Figure S4. Distribution of molecule brightnesses for molecules detected in TAB-PAINT experiments. Data are from three movies of 1 nM Nile blue on MC4100 cells. Frame times were 30 ms. The first and last frame for each molecule was excluded to control for partial 'on' time effects. Frame times were 30 ms. Mean ± standard deviation: 690 ± 380 counts/molecule/frame.

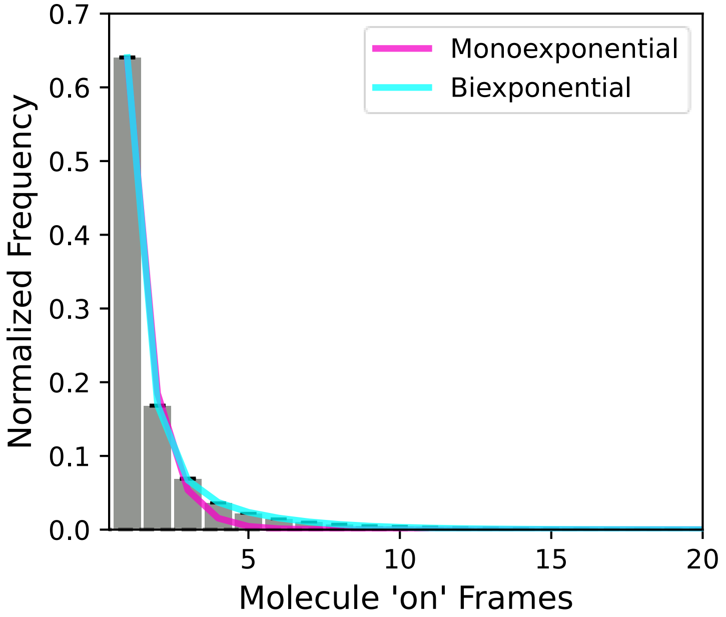

Figure S5. Distribution of 'on' times for molecules detected in TAB-PAINT experiments. Grey bars are averages from three movies with 1 nM Nile blue on MC4100 cells. Error bars are from the standard error of the mean. Frame times were 30 ms. The decay time for the monoexponential fit was 24 ms. Decay times recovered for the biexponential fit were 17 ms and 77 ms with corresponding weights of 0.95 and 0.05, respectively.
